# FACTORS ASSOCIATED WITH SERVICE UTILIZATION AMONG OLDER ADULTS WITH FUNCTIONAL OR COGNITIVE IMPAIRMENT

**DOI:** 10.1093/geroni/igad104.2849

**Published:** 2023-12-21

**Authors:** Yeon Jin Choi, Maria Aranda, Jennifer Ailshire

**Affiliations:** University of Southern California, Los Angeles, California, United States; University of Southern California, Los Angeles, California, United States; University of Southern California, Los Angeles, California, United States

## Abstract

Social services (e.g., senior centers, adult day care centers) and home health services (e.g., in-home personal care, visiting nurses) have been found to provide numerous benefits, such as opportunities for social interaction, physical activity, cognitive stimulation, as well as personal care and regular medical check-ups for older adults. These services may have greater positive impacts among older adults with disabilities or impairments given their limited mobility and reduced capacities to perform daily tasks. However, as most previous studies have focused on the general population, our understanding of factors associated with service utilization among those with disabilities or impairments is limited. This study examined individual characteristics associated with social and home health service utilization among older adults with functional limitations or cognitive impairments using data from the Health and Retirement Study. Results from logistic regression models suggest that older age and being not married were positively associated with utilization of both social and home health services, while being foreign-born was negatively associated with the outcomes, adjusting for sociodemographic characteristics. Respondents with higher educational attainment tend to use social services more and are less likely to use home health services than those with less than a high school education. Rural residence was negatively associated with the utilization of social services, but not with home health services. Providing information on available services and enhancing access by increasing the number of service providers, transportation options, and financial assistance would promote utilization of supportive services among older adults with functional limitations and cognitive impairments.

